# Building a future with root architecture

**DOI:** 10.1093/jxb/ery390

**Published:** 2018-11-15

**Authors:** Marta Del Bianco, Stefan Kepinski

**Affiliations:** Centre for Plant Sciences, Faculty of Biological Sciences, University of Leeds, Leeds, UK

**Keywords:** Climate change, drought, phenology, root architecture, water resources, water use efficiency

**Unmanaged consumption and climate change are profoundly affecting water resources at a global level. It is therefore vital to understand plant responses to drought and flooding, and to breed crops with greater water use efficiency and resilience. Root system architecture is critically important in this, but understanding how architecture and root function should be manipulated to increase resource capture is complex and must take account of the phenology of crop growth and water availability. This virtual issue brings together fundamental research that is helping drive this agenda forward and sets out key areas of development that are needed.**

Fresh water is a relatively rare resource that is fundamental to our survival. While 70% of the earth is covered in water, only 2.5% of that is freshwater, of which a mere 1% is easily accessible. Yet water is often misused. Moreover, in recent years, the effects of climate change have translated into increasing incidences of drought and flooding, and deteriorating water security (Dai *et al.*, 2004; Kron and Berz, 2007). In an important paper, NASA recently highlighted how unmanaged human consumption and climate change have deeply affected water resources at a global level over just 15 years (Rodell *et al.*, 2018). For example, in California’s Central Valley, where intensive agricultural use has been accompanied by persistent drought, groundwater levels have fallen dramatically. A similar trend due to agricultural pressure was observed in Saudi Arabia. Extremes of water availability will put increasing strain on global agriculture that will be felt most acutely in the Global South (especially Africa, Latin America and developing countries in Asia), where a lack of resources and infrastructure makes responding to harsh environmental conditions all the more difficult (Miyan, 2015).

In this context it is of pivotal importance to understand plant responses to drought and flooding, and to breed crops with greater water use efficiency and resilience in the face of more severe environmental conditions. Root architecture is critical for the uptake of both water and nutrients (Lynch, 2013), but understanding how the multiple parameters that define root system architecture and function contribute to effective resource capture is a complex topic that must take account of the phenology of crop growth and water availability (put simply, when it rains relative to when the crop grows) (Tron *et al.*, 2015).

## Development and physiology of different root types

The root system is composed of diverse root types, which differ in origin, anatomy and function (Atkinson *et al.*, 2014; Box 1). Generally speaking, the primary root is of embryonic origin and is the first to emerge upon germination, anchoring the new seedling to the ground and supplying initial nutrients. Some species, like wheat and rice, carry extra embryonic root primordia that give rise to the so-called seminal roots. The term adventitious root describes all plant roots that form from any non-root tissue, such as junction roots (at the root–shoot junction), crown roots (from nodes below ground), brace roots (from nodes above ground), stem roots (internodes), and hypocotyl roots (Steffens and Rasmussen, 2016). All these root types, including primary roots, can develop lateral roots. The number, length and growth angle of these root classes constitute what is normally called root architecture.

**Box 1. Different types of plant root**

Primary and seminal roots are of embryonic origin. Adventitious roots form from any non-root tissue, such as junction roots (root–shoot junction), hypocotyl roots, brace roots (node above ground), and crown roots (node below ground). All of these root types can form lateral roots.

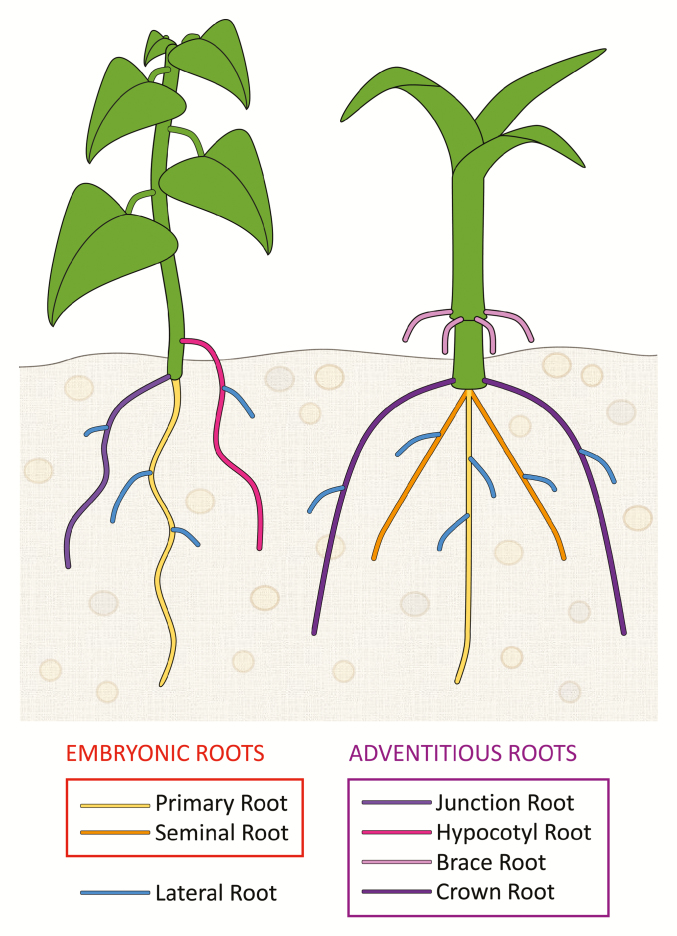

Great effort has gone into deciphering the molecular mechanisms regulating the development of the different root types and their physiological roles. The plant hormone auxin has been shown to regulate the formation of primary, seminal, lateral and adventitious roots [Kircher and Schopfer, 2016 (see also the Insight article by Scheres and Laskowski, 2016); Roberts *et al.*, 2016 (see also the Insight article by Taleski *et al.*, 2016); Herrbach *et al.*, 2017; Du and Scheres, 2018]. Other signalling pathways, involving cytokinin, ethylene, nutrients and stress, interact with auxin to finely regulate root branching and growth (Murphy *et al.*, 2016; Veloccia *et al.*, 2016; Qu *et al.*, 2017). Auxin is also a major regulator of root growth angle (Roychoudhry and Kepinski, 2015; Wang *et al.*, 2018), which is a critical determinant of the distribution of root mass within the soil. Different root types vary in their contribution to structural and absorbance functions, and in terms of the stage of plant growth. Primary and seminal roots are mostly important in the first stages of plant growth. In monocots, seminal and primary roots do not persist at later stages, and the crown and brace roots constitute the majority of the root system (Hochholdinger *et al.*, 2004a, b). In contrast, in dicotyledonous species, lateral roots form the bulk of the root system once the primary root has stopped growing. Transcriptomic work in maize has revealed a possible functional specialization of seminal roots, compared to primary and crown roots, in response to stress (Tai *et al.*, 2016; see also Price, 2016). Interestingly, the presence in maize of mutants lacking only adventitious and seminal roots indicates a common regulatory origin of these two root types (Tai *et al.*, 2017; see also the Insight article by Salvi, 2017). The above-ground brace roots play an important role in structural support of the plant. If they penetrate the soil, brace roots form laterals and help further anchor the plant, while absorbing water and nutrients (Atkinson *et al.*, 2014).

## Environmental modification of root architecture

There is much evidence that water availability can regulate root architecture. Water deficiency in the upper soil suppresses lateral root growth and root growth angle in Arabidopsis (Rellán-Álvarez *et al.*, 2015) and crown root growth in *Setaria viridis* (Sebastian *et al.*, 2016). Flooding, on the other hand, promotes adventitious root formation in rice and elongation in Arabidopsis (Lin and Sauter, 2018). The genetic responses to drought and flooding are also very complex and involve variations in both the transcriptome (Janiak *et al.*, 2016; Kwasniewski *et al.*, 2016; Opitz *et al.*, 2016) and the methylome (Chwialkowska *et al.*, 2016), representing changes in gene expression over both short- and longer timescales. Different cell types respond differently to water status (Opitz *et al.*, 2016), although root hairs seem to be the prime site of water availability perception (Kwasniewski *et al.*, 2016). Responses to variations in water availability involve auxin (Ma *et al.*, 2017; Nakajima *et al.*, 2017), cytokinin (Xu *et al.*, 2016), H_2_O_2_ (Giuliani *et al.*, 2005; Ma *et al.*, 2017), ABA (Kong *et al.*, 2016) and ethylene (Ali and Kim, 2018). Flooding and drought can affect different crop species in distinct ways (Striker and Colmer, 2017; Pavlović *et al.*, 2018). In particular, structural differences, such as number of xylem bundles (Prince *et al.*, 2017; see also Considine *et al.*, 2017) or crown roots (Gao and Lynch, 2016; see also the Insight article by Hochholdinger, 2016) for drought, and root porosity for flooding (Striker and Colmer, 2017), are major components of such variation. From a molecular point of view, auxin has been revealed to be required for hydrotropism in pea and rice, but not in *Lotus japonica* (Nakajima *et al.*, 2017).

## Crop improvement

The central importance of root systems in the acquisition of water and nutrients by plants has meant that they have become a focus of plant breeders and crop improvement programmes (Box 2). In particular, traits such as root length, branching and growth angle determine the distribution of root surface area within the soil profile where nutrients and water are unevenly distributed (Lynch, 2011). Based on a comprehensive experimental analysis, an ideotype for enhanced water and nitrate acquisition has been proposed comprising of a deep-growing primary root with few laterals, steep-angled seminal roots with few long laterals, and crown roots with many laterals (Lynch, 2013). Steeper roots have already been shown to improve water uptake in a number of species including wheat (Manschadi *et al.*, 2006), and nitrogen uptake in maize (Lynch, 2018; see also Varshney *et al.*, 2018). Deeper rooting has also been shown to enhance the yield of rice grown under drought conditions (Uga *et al.*, 2013).

**Box 2. Plant root idiotypes**

External factors, such as water and nutrient availability, can regulate the formation of lateral and adventitious roots, and differential root growth to favour a shallower or deeper root phenotype. While nutrients are often more abundant in the superficial layers of the soil, water can often only be found in the deeper layers, especially during drought.

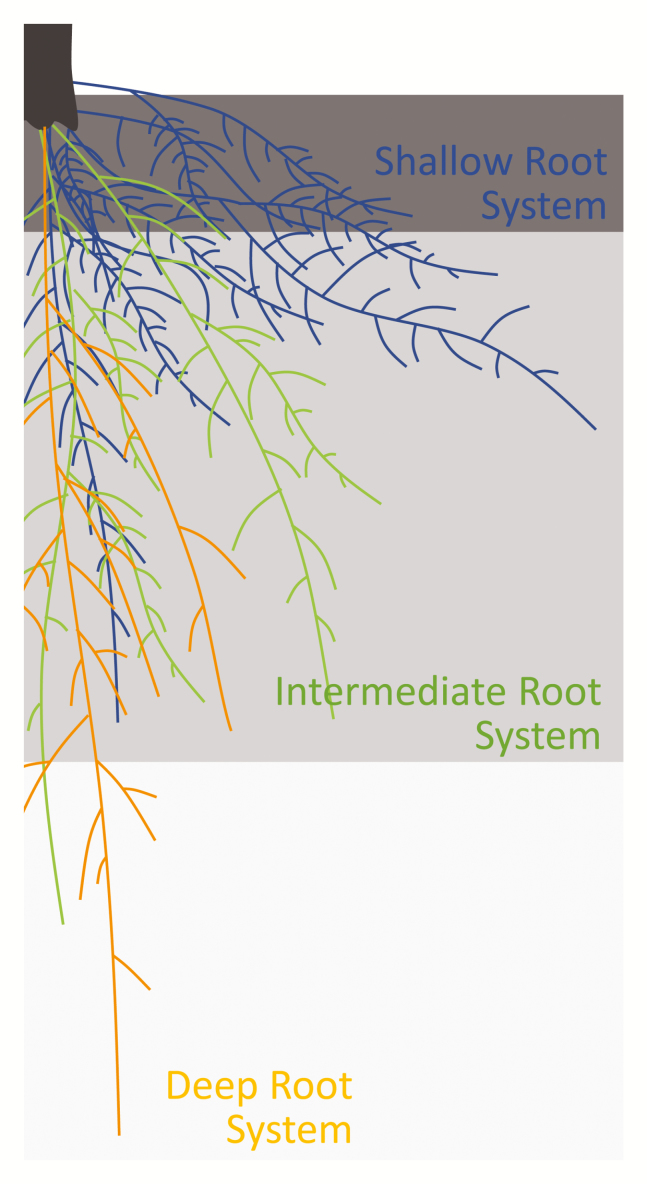

Plant breeding relies on germplasm collections for each crop. These have been described in various species (see also Rebetzke, 2016), including wheat (Lilley and Kirkegaard, 2016; Kenobi *et al.*, 2017), soybean (Prince *et al.*, 2017; Valliyodan *et al.*, 2017), chickpea (Chen *et al.*, 2017), *Lupinus angustifolius* (Chen *et al.*, 2016) and rice (Mori *et al.*, 2016). Phenotyping is a key challenge for plant breeding and technological advances are crucial. In this context, computational approaches represent a valuable means to process, analyze and understand the complexity of root architecture and rhizosphere processes. Researchers have developed a non-invasive platform for maize root and shoot phenotyping able to predict drought tolerance under field conditions by measuring growth parameters at the seedling stage (Avramova *et al.*, 2016). A density-based model, developed in *Brassica rapa*, is capable of reconstructing the growth parameters without the need to perform time-course experiments (Kalogiros *et al.*, 2016). Further, a computational model has been developed in pea to predict mature root characteristics by linking them to genotypes (Zhao *et al.*, 2017). Such approaches represent potentially powerful tools to dissect the developmental biology of root system architecture across a range of species.

Much more field data will be required to tune computational models for plants grown in the field. Fortunately, an advanced imaging system has been developed for the high-throughput phenotyping of root architecture in soil-grown wheat (Wasson *et al.*, 2016). All high-throughput experiments, both *in vitro* and on soil, would benefit from multivariate analysis tools like the one developed for wheat. Phenotyping in combination with the analysis of quantitative trait loci (QTL) has been successful in identifying novel regulators of root architecture in both maize (Salvi *et al.*, 2016) and wheat (Maccaferri *et al.*, 2016). Furthermore, genome editing has opened up the possibility to quickly translate the knowledge gained from fundamental research into improved pre-breeding material and, hence, crops.

## Perspectives

Humans have been selecting crops in some form since the beginnings of plant domestication, some 10,000 years ago. In that time, breeding has completely transformed crops like corn, wheat and banana, and created multiple varieties from wild-type ancestors. Crops have been bred for their productivity or to enhance certain traits like colour or root storage. Importantly, much of the breeding over the last 70 years has been for performance at relatively high levels of agricultural input, including fertilizer application and irrigation (Lynch, 2007). As well as being costly for farmers in financial terms, these inputs are also often associated with a high carbon footprint, something that is incompatible with the concept of the sustainable intensification of agriculture. To meet the challenge of producing more food while minimizing the damage to the very environmental systems within which that food is produced has meant that crop improvement programmes are now heavily focused on new ways to enable crops to perform well with lower levels of inputs and to withstand harsh conditions such as prolonged drought and flooding (Ashraf, 2010; Mustroph, 2018). With this in mind, plant scientists of every kind, from those researching fundamental aspects of plant growth and development to those finding innovative ways to exploit new discoveries in maximising agricultural productivity and minimising environmental harm, are playing their part in giving the planet, and all of its human inhabitants, the greatest chance of making it through the coming century in the best shape possible.
